# Effect of endogenous microbiota on the molecular composition of cloud water: a study by Fourier-transform ion cyclotron resonance mass spectrometry (FT-ICR MS)

**DOI:** 10.1038/s41598-019-44149-8

**Published:** 2019-05-21

**Authors:** Angelica Bianco, Laurent Deguillaume, Nadine Chaumerliac, Mickaël Vaïtilingom, Miao Wang, Anne-Marie Delort, Maxime C. Bridoux

**Affiliations:** 1 Université Clermont Auvergne, CNRS Laboratoire de Météorologie Physique, F-63000 Clermont-Ferrand, France; 2 CEA, DAM, DIF, F-91297 Arpajon, France; 3 Université Clermont Auvergne, CNRS, SIGMA-Clermont, Institut de Chimie de Clermont-Ferrand, F-63000 Clermont-Ferrand, France; 4 Laboratoire de Recherche en Géosciences et Energies (LaRGE), Departement of Physics, Université des Antilles, Pointe-à-Pitre, France

**Keywords:** Atmospheric chemistry, Carbon cycle

## Abstract

A cloud water sample collected at the puy de Dôme observatory (PUY) has been incubated under dark conditions, with its endogenous microbiota at two different temperatures (5 and 15 °C), and the change in the molecular organic composition of this sample was analyzed by Fourier transform ion cyclotron resonance mass spectrometry (FT-ICR MS). Microorganisms were metabolically active and strongly modified the dissolved organic matter since they were able to form and consume many compounds. Using Venn diagrams, four fractions of compounds were identified: (1) compounds consumed by microbial activity; (2) compounds not transformed during incubation; (3) compounds resulting from dark chemistry (i.e., hydrolysis and Fenton reactions) and, finally, (4) compounds resulting from microbial metabolic activity. At 15 °C, microorganisms were able to consume 58% of the compounds initially present and produce 266 new compounds. For this cloud sample, the impact of dark chemistry was negligible. Decreasing the temperature to 5 °C led to the more efficient degradation of organic compounds (1716 compounds vs. 1094 at 15 °C) but with the less important production of new ones (173). These transformations were analyzed using a division into classes based on the O/C and H/C ratios: lipid-like compounds, aliphatic/peptide-like compounds, carboxylic-rich alicyclic molecule (CRAM)-like structures, carbohydrate-like compounds, unsaturated hydrocarbons, aromatic structures and highly oxygenated compounds (HOCs). Lipid-like, aliphatic/peptide-like and CRAMs-like compounds were the most impacted since they were consumed to maintain the microbial metabolism. On the contrary, the relative percentages of CRAMs and carbohydrates increased after incubation.

## Introduction

Microorganisms modify the chemical composition of cloud droplets by using dissolved organic matter (DOM) as a substrate for metabolic activity^1,2^. Cloud microorganisms are able to transform short-chain carboxylic acids into dicarboxylic acids but also formic and acetic acids^3–5^, formaldehyde and methanol^6^. These compounds are used as metabolites to maintain energy levels through the production of adenosine tri-phosphate (ATP) and synthesize larger molecules and create biomass^2^.

In addition, cloud water microorganisms are known to produce pyruvate from lactate^3^ and decompose hydrogen peroxide through their oxidative stress metabolism which, in turn, reduces the oxidative capacity of cloud water^7,8^. These studies suggest that biotic processes compete with photochemical reactivity^9–11^ in the transformation and fate of cloud DOM. However, only few studies have investigated its effect on cloud chemistry focusing on low molecular weight compounds and using isolated strains or endogenous microbiota^5,6,12^.

The cloud aqueous phase contains a complex mixture of organic compounds^13^, including larger multifunctional structures with a substantial fraction of heteroatoms (*e.g*., N, S, and O) expressed in multiple functionalities, such as hydroxyl, carboxyl and carbonyl groups or sulfonate and nitro groups^14–17^. Carboxylic acids and carbonyls have been investigated in detail^18–21^ and, more recently, the presence of amino acids belonging to proteinaceous matter was highlighted in cloud water samples^22^. However, a large fraction (10–50%) of the cloud DOM remains uncharacterized despite the application of various analytical approaches^18,19,23–25^.

A fairly recent approach consists of infusing the whole preconcentrated and desalted cloud DOM sample into the ionization source with an ultrahigh resolution Fourier transform ion cyclotron mass spectrometer (FT-ICR MS). This nontargeted approach allows for the identification of the molecular formula C_*c*_H_*h*_N_*n*_O_*o*_S_*s*_ in multiple compounds and allows for the computation of many useful parameters, such as elemental O/C (oxygen to carbon) and H/C (hydrogen to carbon) ratios^26^, the DBE (double bond equivalent) and the aromaticity index, which are useful parameters for estimating the carbon oxidation state, number of unsaturations and presence of aromatic compounds, respectively. Recent studies have used this powerful approach to investigate the molecular composition of cloud waters^26–28^, revealing the high degree of molecular complexity of this medium, with compounds related to both anthropogenic and biogenic sources in different oxidation states. For example, Cook *et al*. showed the influence of biogenic, urban and wildfire emissions on the molecular composition of cloud water samples collected at Whiteface Mountain (US)^28^, while Bianco *et al*. found a large contribution of biologically derived materials, such as lipids, peptides and carbohydrates, in cloud waters sampled at the puy de Dôme (PUY) station (France)^27^.

The main objectives of this study were (1) to evaluate variations in cloud DOM molecular diversity based on the activity of endogenous microflora in clouds and (2) to study how temperature can modulate this biological response. For this study, cloud water collected at PUY was incubated at two temperatures and analyzed by FT-ICR MS. Cloud processing is known to contribute to secondary organic aerosol (SOA) generation^10,25,29^, and this work will help to evaluate the impact of microorganisms on cloud organic matter transformations.

## Results

Cloud water was collected on June 1^st^, 2016, at the PUY station, between 2:50 pm and 7:20 pm. The air mass origin and FT-ICR MS analyses of this sample were discussed in a previous work^27^. This study showed that the puy de Dôme summit was located in the free troposphere during sampling, as indicated by the LACYTRAJ back-trajectory model. The sample was classified as marine using a multicomponent statistical analysis of the aqueous inorganic composition, as presented in Deguillaume *et al*.^20^. Continental influence may, however, not be excluded because the air mass was located below the boundary layer before arriving to the puy de Dôme station. Chemicophysical and microbiological characterization are reported in Supplementary Table S1. This table also reports the relative degradation or production of targeted chemical compounds in BIO + CHEM 5 and 15. The total number of cloud microorganisms was 8.6 × 10^4^ cells mL^−1^, as measured by the flow cytometry and microbial activity, which were confirmed by the ATP measurement (2.24 pmol mL^−1^). These values show that the microbial characteristics of this cloud sample are consistent with those in previous studies conducted on cloud water samples^7^.

The cloud water sample was filtered, preconcentrated on a solid phase extraction (SPE) cartridge and analyzed by FT-ICR MS. This fraction is referred to as “INITIAL” in the rest of the article. More detailed information about the analysis is given in the materials and methods section and in Supplementary S(1). Incubation experiments using the endogenous microbiota were then performed in the lab. Supplementary Fig. S1 summarizes all of the incubation tests that have been performed in this study. Cloud water was incubated in the dark, which allowed us to investigate the effects of dark chemistry (i.e., hydrolysis and Fenton reactions) and microbial metabolism. Two fractions containing endogenous microbial populations were incubated at 15 °C (BIO + CHEM 15) and 5 °C (BIO + CHEM 5) and preconcentrated and analyzed by FT-ICR MS with the same procedure used for INITIAL. Values of 5 °C and 15 °C were chosen as representative temperatures measured at the PUY station in winter and summertime, respectively. Transformations of the DOM values observed for these two fractions are attributed to both microbial metabolism and dark chemistry. To evaluate the contribution of dark chemistry only, two fractions of cloud water (CHEM 15 and CHEM 5) were also filtered to eliminate microorganisms, incubated in the same conditions as those used for BIO + CHEM 15 and 5 and analyzed by FT-ICR MS using the same set of experimental parameters. By comparing the compositions of BIO + CHEM and CHEM, the transformations resulting from microbiological processes only are highlighted. In terms of the acquisition of sequential mass windows, relative abundance was not considered in this study. In the first approach, the formation and degradation of organic compounds were evaluated on the basis of the comparison/disappearance of peaks (and associated assigned molecular formulas).

### Van Krevelen diagrams: effects of microbial and chemical transformations

**Figure 1:**
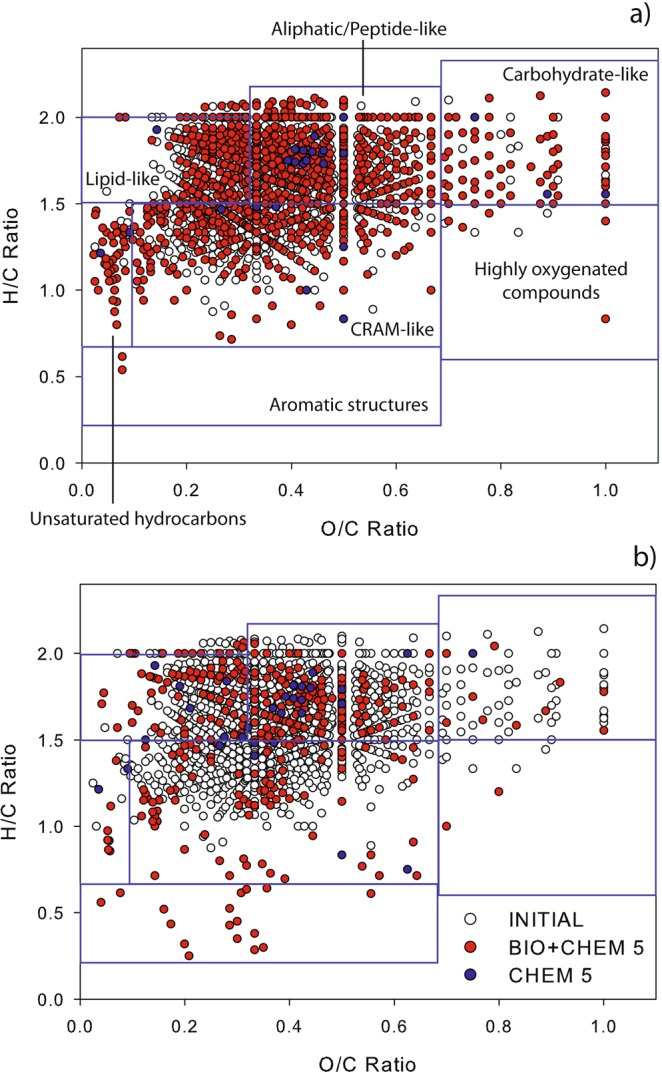
VK diagrams of the INITIAL (white) and incubated fractions, with microorganisms (BIO + CHEM, red dots) at 15 °C (**a**) and 5 °C (**b**). The CHEM fraction is represented in both plots by the red and blue dots.

Figure 1a shows van Krevelen (VK) diagrams corresponding to INITIAL, BIO + CHEM 15 and CHEM 15. Incubation at the highest temperature causes the degradation of a large fraction of compounds but also the formation of many new compounds. Figure 1a suggests the negligible impact of dark chemistry on the molecular composition of the sample. Indeed, the CHEM 15 (in blue in Fig. 1a) experiment did not result in any compound degradation, and only 25 compounds were produced when compared to the INITIAL sample. Since the dark chemistry contribution is non-significant, the result of the BIO + CHEM 15 incubation mainly reflects endogenous microbial metabolism. This incubation (BIO + CHEM 15) results in a significant decrease (42%) in the total number of compounds detected when compared to the INITIAL sample (1084 in BIO + CHEM 15 vs. 1887 in INITIAL).

Assigned molecular formulas were subdivided into four subgroups based on their elemental compositions: CHO (containing only C, H and O), CHNO (containing C, H, N and O), CHOS (containing C, H, O and S) and CHNOS (containing C, H, O and both N and S) (Supplementary Fig. S2). Incubation without microorganisms (CHEM) did not result in any significant variation in the abundance of the relative subgroup (less than 2.3%), except for CHNOS abundance, which displayed a 2.4% increase. Supplementary Figs S3 and S4 report the average values of various parameters calculated for the two incubations and INITIAL, respectively, at 15 °C and 5 °C, including the median DBE values (indicating the total number of π bonds plus rings), O/C, H/C, OSC (carbon oxidation state, a parameter expressing the oxidation degree of atmospheric organic compounds; see Supplementary Fig. S(2)), and AI (aromaticity index, a criterion used to highlight the presence of aromatic cores; see Supplementary Fig. S(3)). The CHO index, describing the relative H, O, and C contents in the assigned molecular formula, has previously been used by Mann *et al*.^30^ (Supplementary Fig. (S4)); higher CHO index values are attributed to more oxidized compounds, while lower values indicate reduced molecules. At first glance, the average values of O/C and H/C do not vary significantly. However, compounds with O/C ≥ 0.5 and H/C ≥ 1 represent higher relative abundances (24.4%) for BIO + CHEM 15 than INITIAL (18.5%). This result suggests the production of oxidized compounds with O/C > 0.5 during incubation at 15 °C.

The analysis of the BIO + CHEM 5 and CHEM 5 results, compared with those of the BIO + CHEM 15 and CHEM 15 fractions, demonstrates the influence of temperature on endogenous cloud microbial activity. As observed for incubation at 15 °C, many compounds are degraded and formed, but some compounds are specifically produced at 5 °C. The effect of dark chemistry on molecular composition is minor: only 27 compounds are formed, which are shown in blue in Fig. 1b. The incubation of microorganisms at 5 °C leads to the production of a lower number of new compounds compared with the incubation experiment at 15 °C. Indeed, BIO + CHEM 5 contains 374 compounds, corresponding to approximately 20% of the initial number of assigned molecular formulas. In addition, produced compounds are different from the compounds produced during incubation at 15 °C: 190 compounds are common, and 173 are specifically formed at 5 °C. The average H/C values decrease by 7% at 5 °C, and this result is not observed at 15 °C (Supplementary Fig. S4). This could be explained by the presence of 35 compounds with O/C ≤ 0.5 and H/C ≤ 1 in the incubated fraction (9.4% of the total assigned formula), while INITIAL contains only 9 compounds (0.5%).

### Venn diagram: incubation at 15 °C

**Figure 2:**
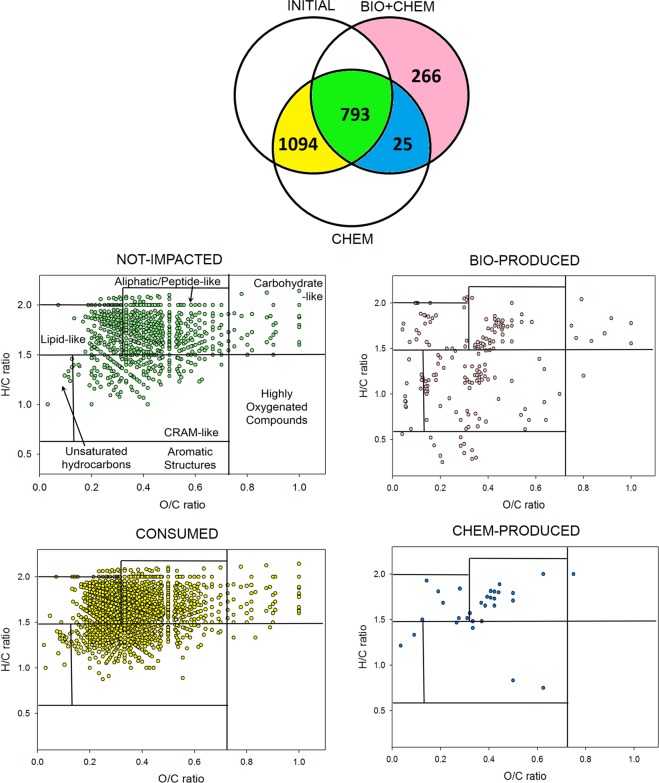
Top panel: Venn diagram of the INITIAL, BIO + CHEM and CHEM fractions for incubation at 15 °C. The yellow area represents compounds consumed by microorganisms (CONSUMED); the green area (NOT IMPACTED) corresponds to compounds not modified by chemical and biological processes; the blue area displays compounds produced by dark chemistry reactions (CHEM-PRODUCED); the pink area represents compounds produced by microorganisms (BIO-PRODUCED). The VK diagrams corresponding to each colored area of the Venn diagram are displayed below using the same color code.

The Venn diagrams^31^ are drawn in Fig. 2 to quantify the effect of microbial activity on the cloud chemical composition. This allows for the comparison of compounds contained in INITIAL_,_ BIO + CHEM 15 and CHEM 15 since these diagrams represent the number of compounds that are either shared or uniquely expressed in each of the three samples. The yellow area (CONSUMED) contains compounds transformed by microbial activity since they are present in INITIAL and CHEM 15 and not in BIO + CHEM 15. The green area (referred to as NOT-IMPACTED) shows the compounds that remain unaltered by the incubation experiments. The blue area (CHEM-PRODUCED) displays the compounds shared by CHEM 15 and BIO + CHEM 15 but not INITIAL. These compounds are produced by hydrolysis or dark reactions (i.e., Fenton reactions). The pink area (BIO-PRODUCED) contains compounds that are only present in BIO + CHEM 15, thus resulting from microbial transformations. A total of 1084 compounds are consumed by the microorganisms, and 266 are produced, while 793 are not impacted (i.e., partially produced or degraded but without the disappearance of the mass peak) by microbial activity. Figure 2 also reports the VK diagrams corresponding to the CONSUMED, NOT-IMPACTED, CHEM-PRODUCED and BIO-PRODUCED compounds, highlighting their distribution among the major biochemical classes (i.e., lipids, proteins, lignins, carbohydrates, and condensed aromatics) (Supplementary Table S2).

### Effect of microbial activity: comparison of consumed and bio-produced

**Figure 3:**
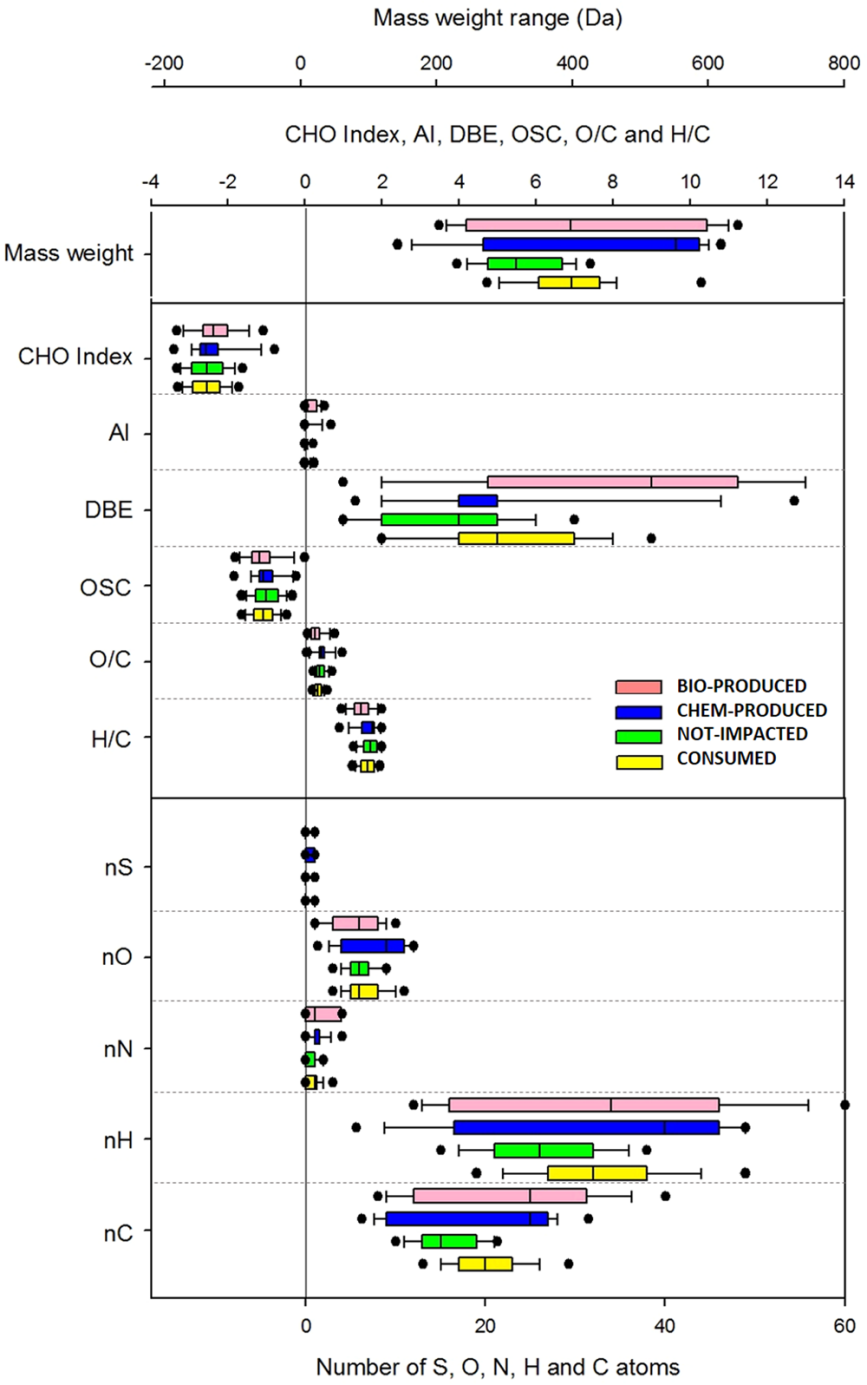
Box plots of the molecular weight, CHO index, AI, DBE, OSC, O/C, H/C and number of atoms calculated for the different fractions (BIO-PRODUCED, CHEM-PRODUCED, NOT-IMPACTED, and CONSUMED) reported in Fig. 2 for incubation at 15 °C. The bottom and top lines of the boxes correspond to the 25^th^ and 75^th^ percentiles, respectively. The middle line represents the median. The ends of the whiskers represent the 10^th^ and 90^th^ percentiles, and the filled circle denote the outliers. The y-axis shows the parameters; the bottom x-axis reports the values for nS, nO, nN, nH and nC; the top x-axis represents the values for the CHO index, AI, DBE, OSC, O/C, and H/C, while the uppermost x-axis reports the molecular weight values.

Figure 3 reports the average number of carbon (nC), nitrogen (nN), hydrogen (nH), oxygen (nO) and sulfur (nS) atoms for CONSUMED, NOT-IMPACTED, CHEM-PRODUCED and BIO-PRODUCED. The NOT-IMPACTED compounds have significantly lower nC, nH and mass weight in comparison to the CONSUMED, CHEM-PRODUCED and BIO-PRODUCED compounds. The focus of this work deals with the effects of microbial degradation on organic matter; thus, the comparison between CONSUMED and BIO-PRODUCED is further analyzed. The carbon number (nC) in BIO-PRODUCED is higher than that in CONSUMED, and the values are more dispersed; the average nH does not significantly vary, while the nN and nS values increase by more than 10% compared to the CONSUMED values. The oxygen number (nO) decreases slightly with the weakening of O/C during incubation. Figure 3 also shows the median values of DBE, O/C, H/C, OSC, and AI. The variation significance was checked with the t-test. The H/C, O/C, OSC, AI and CHO indexes do not vary significantly. Surprisingly, the DBE median value in BIO-PRODUCED is higher than that in CONSUMED. The ranges of observed molecular weights are not significantly different between the CONSUMED and BIO-PRODUCED fractions, but the values are more dispersed in BIO-PRODUCED.

### Division into classes

The VK diagram can be divided into seven categories (Supplementary Table S2) based on the stoichiometric ranges of O/C and H/C corresponding to the seven classes of compounds found in natural organic matter^27^: (1) lipid-like, (2) aliphatic/peptide-like, (3) carboxylic-rich alicyclic molecule (CRAM-like), (4) carbohydrate-like, (5) unsaturated hydrocarbon, (6) aromatic and (7) highly oxygenated compound (HOC) structures. CRAMs consist of carboxylated alicyclic structures and contain structures that are similar to large, fused, non-aromatic rings, with a high ratio of substituted carboxyl groups. Peptides are biomolecules consisting of short chains of amino acid residues. Lipid-like materials include fats, waxes, sterols, fat-soluble vitamins, monoglycerides, diglycerides and triglycerides. All of these molecular families are related to biological activities. Carbohydrate-like materials are complex polymeric structures composed of acyl polysaccharides, which contain varying levels of carbohydrate, lipid and acetate groups. HOCs are organo-nitrates and nitro-oxy organosulfates previously detected in aerosols and fog water samples^32–37^.

**Figure 4:**
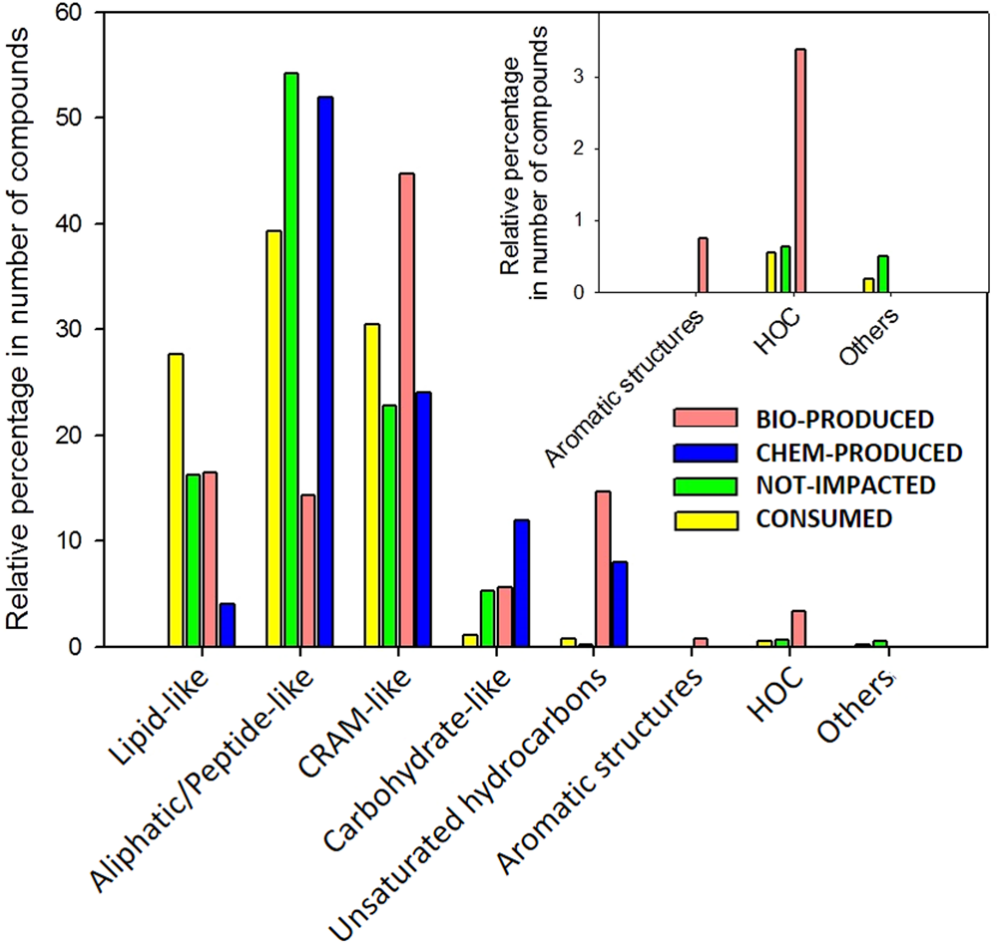
Relative abundances of compounds contained in lipid-like, aliphatic/peptide-like, CRAM-like, carbohydrate-like materials; unsaturated hydrocarbons; aromatic structures and HOCs in CONSUMED, NOT-IMPACTED, BIO-PRODUCED and CHEM-PRODUCED fractions for incubation at 15 °C.

A detailed composition of INITIAL has been previously described and discussed in Bianco *et al*.^27^ and compared with other samples collected at PUY and Stormpeak Laboratory (US) by Zhao *et al*.^26^. Compounds contained in CONSUMED, BIO-PRODUCED, CHEM-PRODUCED and NOT-IMPACTED were analyzed following the same approach. Figure 4 presents the relative percentages of each class in the number of compounds. All of the classes are impacted by microbial transformations, with either an increase or decrease in the number of compounds. Endogenous microbiota preferentially degrade lipid-like and aliphatic/peptide-like materials. For lipid-like materials, the number of compounds decreases drastically from 303 to 44 in the assigned molecular formula; furthermore, the average weight of the molecular compounds in this class increases significantly after incubation (average values of CONSUMED = 398 ± 79 Da and BIO-PRODUCED = 540 ± 129 Da) (Supplementary Table S3). The number of molecular formulas assigned to the aliphatic/peptide-like class also strongly decreases from 430 (39.3% of the total) to 38 (14.3%). The CRAM-like compounds are produced during incubation. Surprisingly, incubation leads to the formation of unsaturated hydrocarbons (from 8 to 39 molecular assigned formulas) and aromatic compounds (formation of 2 molecules).

### Comparison between incubations at 5 °C and 15 °C

**Figure 5:**
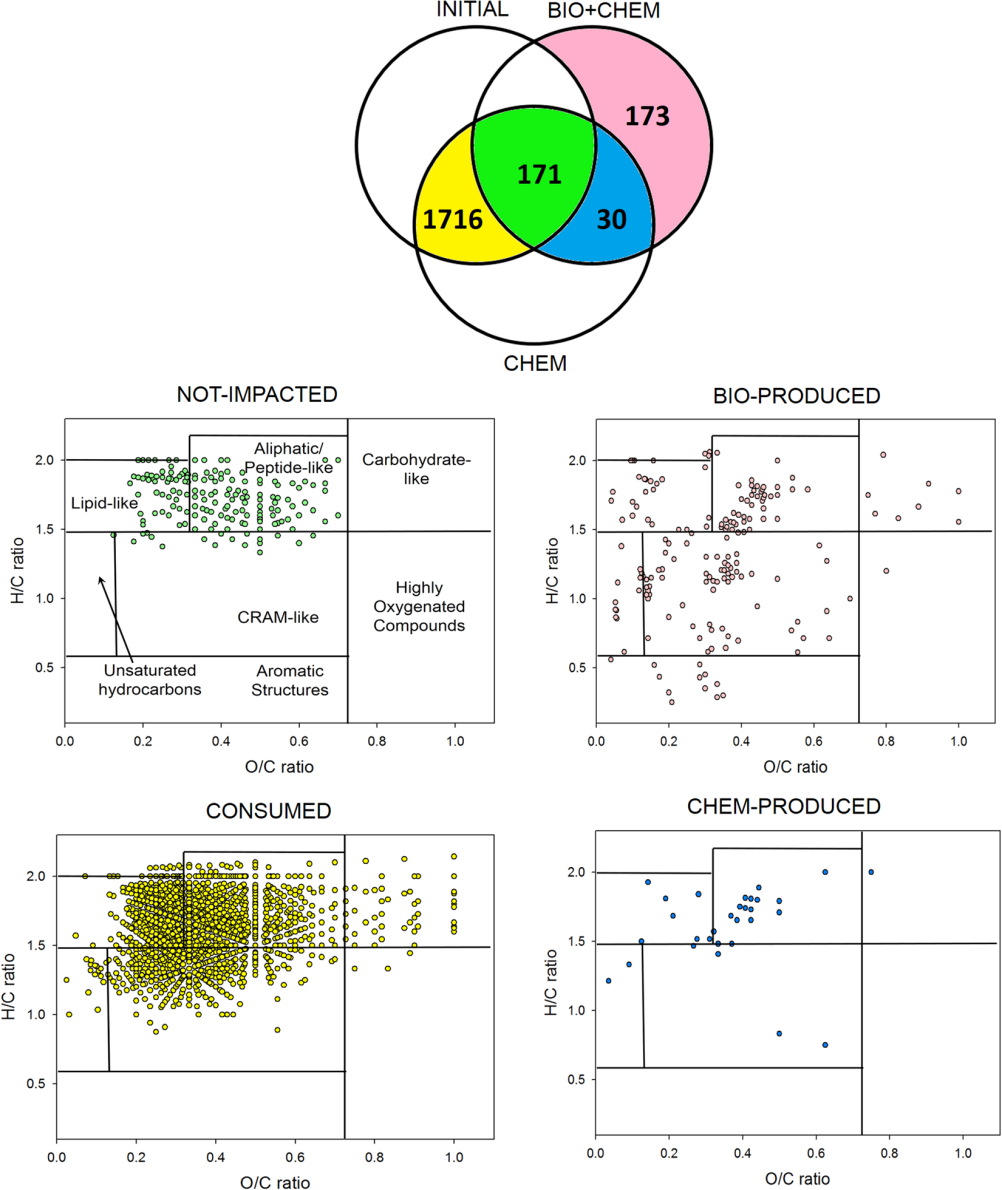
Top panel: Venn diagram of the INITIAL, BIO + CHEM and CHEM fractions for incubation at 5 °C. The yellow area represents compounds consumed by microorganisms (CONSUMED); the green area (NOT IMPACTED) corresponds to compounds not modified by chemical and biological processes; the blue area displays compounds produced by dark chemistry reactions (CHEM-PRODUCED); the pink area represents compounds produced by microorganisms (BIO-PRODUCED). The VK diagrams corresponding to each colored area of the Venn diagram are displayed below using the same color code.

A similar analysis was also performed for incubation at 5 °C. Figure 5 displays the Venn diagram and VK plot in the same way as Fig. 2. A comparison with the incubation at 15 °C reveals that a higher number of compounds is degraded (1716 at 5 °C instead of 1094 at 15 °C), but a lower number is produced (173 at 5 °C instead of 266 at 15 °C). As observed for incubation at 15°C, the molecular assigned formulas are distributed among the seven classes of compounds found in natural organic matter, except for NOT-IMPACTED, where the compounds are clustered in lipid-like and aliphatic/peptide-like regions. In the following paragraph, the comparison between CONSUMED and BIO-PRODUCED is detailed.

BIO-PRODUCED median values of nC, nH and nO increase in comparison to CONSUMED values (Supplementary Fig. S5). As observed earlier, H/C, O/C and OSC do not vary significantly, while the DBE values are higher for BIO-PRODUCED compared with the values calculated from CONSUMED. The comparison between the BIO-PRODUCED groups for incubations at 5 and 15 °C shows that median DBE values do not vary significantly, but incubation at 5 °C leads to the production of molecules associated with higher DBE values (75^th^ percentile of approximately 16 at 5 °C and approximately 11 at 15 °C). Interestingly, the AI average value increases by 0.2 units (from 0.03 to 0.23), while it increases by 0.15 units for incubation at 15 °C. This may be explained by the production of a larger number of compounds in the lower part of the VK diagram (O/C < 0.67 and H/C ≤ 0.67). The median value of the molecular weight is higher for BIO-PRODUCED compared to CONSUMED. The comparison of BIO-PRODUCED between 15 and 5 °C shows that the molecular weight after incubation increases more at 5 °C than at 15 °C (26% instead of 6% of the average value).

Supplementary Figure S6 displays the relative abundances of compounds contained in the seven classes of compounds found in the DOM for the incubation experiments conducted at 5 °C. A decrease of 10.5% is observed in the relative percentage of the lipid-like compounds, and the mass weight decreases from 387 ± 81 to 333 ± 60 Da (Supplementary Table S3). In the aliphatic/peptide-like class, the average molecular weight (from 397 ± 89 to 555 ± 96 Da) increases with the formation of a molecular formula with more than 20 carbon atoms. The molecular weight is also higher for CRAM-like compounds (increase of 157 Da). As observed for incubation at 15 °C, compounds belonging to unsaturated hydrocarbon and aromatic classes are produced during incubation; in particular, incubation at 5 °C leads to the production of 17 compounds.

## Discussion

These experimental results clearly highlight that endogenous microbiota alter the molecular composition of DOM in cloud water through the degradation of many compounds and the production of many others, while transformations by dark chemistry are negligible. Our results are consistent with those in previous experiments performed on three different cloud samples collected at the same site, with similar chemical and biochemical compositions^7^. In this study, only very limited numbers of short chain carboxylic acids (acetate, formate, succinate, oxalate and malonate), formaldehyde and hydrogen peroxide were considered, and microbial activity was largely prevailing with respect to dark chemistry. In the present work, we extended the investigation to a wider number of compounds presenting much larger molecular weights and more diverse chemical composition. Several hundred organic compounds were completely degraded by cloud microorganisms (1716 at 5 °C, 1094 at 15 °C), and hundreds of new compounds were biosynthesized (173 at 5 °C, 266 at 15 °C). Temperature is a stress factor for microorganisms in the atmosphere: at 5 °C, cell division is slowed down and after incubation the number of cell is lower at 5 °C than at 15 °C. In contrast, at low temperature, microorganisms need more energy to maintain metabolical activity and to produce ATP. For this reason, they consume more carbon sources, consuming more compounds during incubation.

One limitation to the present study is that ESI-FT-ICR MS is not a quantitative method. Only mass peaks that completely disappear and newly formed peaks are considered. Thus, the impact of microbiological activity on cloud chemistry is definitely underestimated. Many compound concentrations are changing as a consequence of microbial transformations but without the formation/disappearance of mass peaks. This can be observed regarding the biodegradation of formaldehyde, formate and acetate, which are not fully consumed during incubation (Supplementary Table S1). Even if they are impacted by microorganisms, they are part of the NOT-IMPACTED group following the presented classification. Abiotic transformations by hydrolysis and Fenton processes also partially degrade some compounds, but no variation in the number of compounds is detected. Moreover, solid phase extraction leads to the loss of some low molecular weight compounds.

Microbial metabolism is very complex and includes numerous pathways involved in building up and breaking down cellular components. Microbial extracellular enzymes may transform large molecules into smaller compounds in cloud water. Small molecules, which are initially present in the aqueous cloud phase or produced by aqueous phase reactivity, can be transported through the microbial membrane. Inside a microbial cell, small molecules are used to produce energy, converted into other small molecules or used to synthetize larger molecules. These large molecules, such as DNA or proteins, can be either integrated into the biomass or excreted in the cloud medium. Intracellular microbial metabolism also produces small molecules, which can be exported out of cells. Moreover, cloud microbiota are a dynamic system, where microbial cells are constantly growing or dying. As a consequence, cell lysis can occur and release both large and small compounds in cloud water. The impact of microbial activity on the cloud chemical composition is hard to evaluate since it is able to produce and consume organic compounds. For this reason, the CONSUMED and BIO-PRODUCED compounds may not necessarily be correlated.

Considering incubation at 15 °C, the H/C and O/C ratios do not vary significantly between CONSUMED and BIO-PRODUCED, but microbial transformations lead to the production of some more oxidized and reduced compounds (Fig. 1a,b). The DBE value and number of carbon atoms increase after incubation. The median value of the molecular weight remains constant, but more dispersion is observed with the combined production of both higher and lower weighted compounds. This could be due to the synthesis of high weight molecular compounds from small molecules and excretion out of the microbial cell. For example, cloud microorganisms were shown to produce extrapolymeric substances^12^, biosurfactants^38^ and siderophores^39^. This biological activity is also observed in other environments, such as surface waters^40^. As reported before, the presence of high weight molecular compounds could also be the result of cell lysis.

Cold shock is a stress condition for microorganisms, and microorganisms handle this stress by modulating their metabolism^41^. A recent study conducted on *Pseudomonas syringae* isolated from cloud samples at the PUY station showed the metabolic effects of a temperature change from 17 °C to 5 °C^42^. This bacterium belongs to a more frequent and major active group in clouds^43,44^. *Pseudomonas syringae* synthetized cryoprotectants (namely, trehalose, glucose, glycerol, carnitine and glutamate) as a metabolic response to this cold shock^42^. The lipid metabolism was also altered by changing the saturation level of the fatty acids to preserve the membrane fluidity of the bacterium. In addition, the carbohydrate metabolism was activated to produce more energy (i.e., higher amounts of ATP), and the amino-acid metabolism was modified together with the synthesis and consumption of short di- and tetra-peptides.

In this work, we also observed a number of important changes in the metabolism of the cloud microbiome when incubations were performed at 5 °C compared to 15 °C. For example, incubation at 5 °C leads to the production of a lower number of compounds, which is different from those observed during incubation at 15 °C. Although the DBE median values are quite similar for both temperatures, a net shift toward higher molecular weights and nC is observed at 5 °C, as shown by the increase in the 75^th^ percentile (Fig. 3 and Supplementary Fig. S5). For these reasons, even if a large number of compounds is degraded, many compounds are also biosynthesized. This DBE increase could partially be related to the synthesis of unsaturated lipids to modulate the membrane fluidity in response to a cold shock, which is in agreement with previous reports^42^.

**Figure 6:**
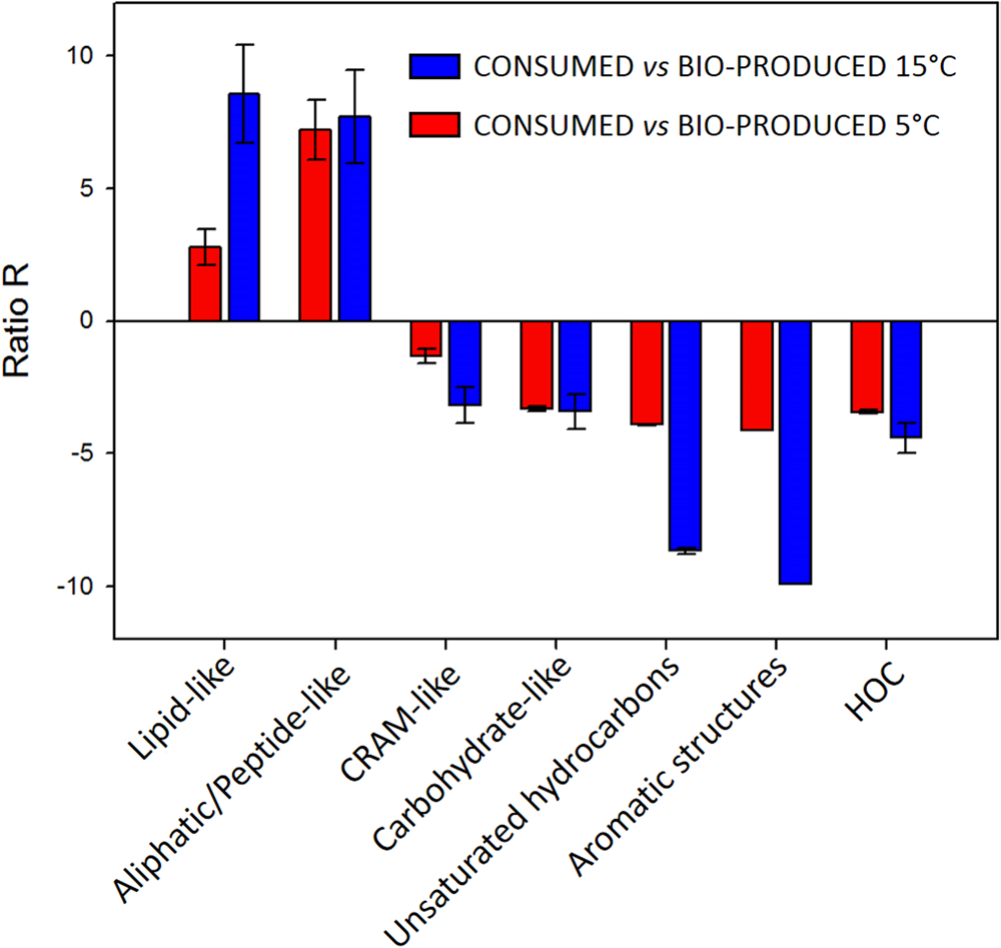
R parameter: comparison between the compositions of each class in CONSUMED and BIO-PRODUCED for incubations at 15 °C (blue) and 5 °C (red).

All chemical classes undergo a decrease in the number of compounds, except for aromatics and unsaturated hydrocarbons, whose numbers increase at both incubation temperatures. In addition, the impact of microbial transformations is different for each class, as highlighted by the parameter R, which is calculated using Eq. E1 (shown in Fig. 6) for both sets of incubations at 5 °C and 15 °C:

E1 $$R=\frac{a}{b}-\frac{c}{d}$$

where *a* = n° compounds in CONSUMED for the considered class, *b* = n° compounds in BIO-PRODUCED for the considered class, *c* = total n° of compounds in CONSUMED and *d* = total n° of compounds in BIO-PRODUCED. For R > 0, compounds are consumed; for R < 0, they are produced. We can observe that even if the number of compounds decreases in each class, lipid-like and aliphatic/peptide-like materials are degraded, while in the other classes, compounds are produced.

Compounds related to the lipid-like class are consumed in both incubations (Fig. 6). These compounds may preferentially be degraded because of the high yield of ATP given by their oxidation, but they can also be incorporated into membranes during new cell formation. It is likely that microorganisms multiply during the incubation time scale, as previously observed by Amato *et al*.^45^. Low molecular weights of lipid-like materials are likely rapidly transformed into short chain carboxylic acids and other oxidized short molecules with great variations in the H/C and O/C ratios. For organic compounds in the lipid-like class with a molecular weight higher than 450 Da, CONSUMED contains 44 assigned molecular formulas, with an average mass of 524 ± 99 Da, H/C = 1.72 ± 0.10 and O/C = 0.24 ± 0.03. BIO-PRODUCED contains 44 assigned molecular formulas, with an average mass weight of 540 ± 129 Da, H/C = 1.72 ± 0.16 and O/C = 0.24 ± 0.04. Microorganisms are not able to degrade these compounds but can oxidize them. The first step of oxidation is usually hydroxylation; this explains the increase in the average mass weight of 16 Da (i.e., one oxygen atom). Thus, high weight lipid-like materials are weakly oxidized, and the small variations in their H/C and O/C values leaves them in the lipid-like class. This could justify the apparent lack of variation in their H/C and O/C ratios. Aliphatic/peptide-like compounds are also used by microorganisms for protein synthesis or as a source of nitrogen. For this class, the average molecular weight decreases, and the compounds are mostly degraded by microorganisms (Fig. 6). Table S3 shows that this class of compounds is degraded more at lower temperature (758 molecular formulas assigned in CONSUMED at 5 °C and 430 at 15 °C). This is likely because microorganisms need more energy to maintain their metabolism at low temperatures. A previous study reported that both amino acids and peptide metabolisms were modified when a cloud bacterium was incubated at a low temperature^42^.

CRAM-like compounds are formed during incubation. They present structural features that are common with those of terpenoids (i.e., membrane constituents and secondary metabolites in a wide range of prokaryotic and eukaryotic organisms^46^). Hertkorn *et al*. reported that CRAM-like compounds are the decomposition products of biomolecules, as indicated by the prevalence of carboxyl groups and the pattern of increasing oxidation with decreasing molecular size^47^. This could explain the production of newly oxidized and smaller compounds in this class. Supplementary Figs S7 and S8 compare O/C vs. molecular weight for CRAM-like compounds in CONSUMED and BIO-PRODUCED at 15 °C and 5 °C, respectively. CONSUMED CRAM-like compounds, with a molecular weight range of 300–400 Da, are degraded to (probably) give BIO-PRODUCED compounds with a molecular weight range of 200–300 Da and a higher O/C. The core of the BIO-PRODUCED assigned molecular formulas, with a molecular weight range of 500–650 Da (right of the plot in Supplementary Figs S7 and S8), may result from the partial oxidation of lignin residues^48,49^, which have high molecular weights and fall in the same area as CRAM-like compounds^50^. Lignin residues are difficult to be ionized using ESI, but biodegradation may introduce oxidation, making them easily detectable. Carbohydrate-like compounds are produced during incubation, and the average molecular weight decreases. This result is consistent with the usual biotransformation of carbohydrate polymers into oligomeric compounds, resulting in short chain molecules. As an example, cellulose is biodegraded in oligosaccharides, resulting in dimers or monomers, which can be uptaken by the cells. The effect of temperature is clear for carbohydrate-like compounds: even though they are produced, a fraction is consumed to produce ATP. This fraction is larger for the incubation at 5 °C (52 compounds) than for the incubation at 15 °C (12 compounds); this is consistent with the activation of the carbohydrate route and the increase in the ATP concentration observed at low temperatures^42^. Saccharides can also be used to produce intracellular trehalose, which is a well-known cryoprotectant^42^.

The formation of aromatics and unsaturated hydrocarbons is also observed (Fig. 6), and this increase is particularly highlighted by the fact that INITIAL does not contain (or contains only a few) molecular assigned formulas in these classes. The presence of aromatics could result from lignin biotransformation, for instance, while unsaturated hydrocarbons are consistent with the increase in unsaturated lipids.

This study clearly indicates that microbial activity is able to strongly modify organic matter in clouds. This leads to the general assumption that cloud microorganisms are able to modify the chemical properties of aerosol particles and, thus, impact atmospheric processes. These processes were shown to synthetize higher molecular weight compounds, such as exopolymeric substances or oligosaccharides, which can interact with water. The cloud microbiota may then affect the formation of cloud condensation nuclei, and surface-active molecules could also be produced or released into the cloud water by cell lysis. These molecules are able to reduce the surface tension level of aerosol particles and could, thus, enhance their ability to be activated into cloud droplets^51–53^. These molecules could also migrate towards the surface phase, reducing hygroscopic water uptake and perturbing the efficiency of the mass transfer between the gas and aqueous phases of clouds. Siderophores could also be produced by microorganisms for the uptake of iron required for their metabolism. Theses complex molecules are able to strongly complex iron in cloud water, changing its redox cycle and role in the cloud oxidative capacity^39,54^.

All of these microbial transformations have been shown to be modulated by temperature. Levels of oxidants, such as hydrogen peroxide, are also key parameters controlling the complex and highly variable cloud microbiome metabolism^8^. This demonstrates the necessity of performing similar high-resolution mass spectrometry investigations on other incubations of various cloud samples with contrasted chemical and biological compositions. Cloud waters will be collected at very different geographical sites, exposed to different environmental conditions. It will be also important to combine photo- and biodegradation processes during incubation experiments to evaluate their potential synergic effect occurring simultaneously in clouds. A targeted analysis will be performed in parallel on the selected organic compounds strongly transformed during the incubations to assess the mechanisms and their modulations by microorganism.

## Experimental Materials and Methods

The PUY station belongs to European atmospheric survey networks: ACTRIS (Aerosols, Clouds, and Trace gases Research Infrastructure) and EMEP (the European Monitoring and Evaluation Program). The PUY observatory also belongs to the GAW (Global Atmosphere Watch) stations. A dynamic one-stage cloud water impactor (cut off diameter of approximately 7 µm^55,56^) was used to sample the cloud droplets. Before cloud collection, the aluminum impactor was cleaned and sterilized by autoclaving. The sample was stored in sterilized bottles; a fraction of the cloud water was filtered using a 0.22 µm nylon filter within 10 min after sampling to eliminate particles and microorganisms. The hydrogen peroxide concentration and pH were determined a few minutes after sampling. A fraction was frozen on site and stored in appropriate vessels at −25 °C until these samples were analyzed to estimate the ion concentrations by ion chromatography (IC), total organic carbon (TOC) by a TOC Shimadzu analyzer and iron concentration by the spectrophotometric method^57^. More details about the physicochemical analysis are reported in Bianco *et al*.^27^. Microbiological analyses were performed on nonfiltered cloud samples. Cell counts were performed by flow cytometry (BD FacsCalibur, Becton Dickinson, Franklin Lakes, NJ) on 450 μL triplicates, which were added with 50 μL 5% glutaraldehyde (0.5% final concentration; Sigma-Aldrich G7651) stored for <1 week at 4 °C. For analysis, the samples were mixed with 1 vol. of 0.02 μm filtered Tris-EDTA, with a pH of 8.0 (40 mM Tris-Base; 1 mM EDTA; acetic acid to pH of 8.0), and stained with SYBRGreen I (Molecular Probes Inc., Eugene, OR) from a 100X solution. The counts were performed for 3 min (or 100,000 events) at a flow rate of ~80 μL min^−1^ (precisely further determined by weighting). The ATP concentration was determined using the following procedure: 400 µL of the sample was strongly mixed in a microtube with 400 µL of the extractant B/S from the ATP measurement kit used (ATP Biomass Kit HS, Biothema) and stored in a frozen state until further analysis. The ATP concentrations were determined by bioluminescence^58^, as reported by Amato *et al*.^3^.

The cloud water sample was treated and analyzed by FT-ICR MS, filtering the signal by S/N > 5. In this way, mass peaks with low intensity (lower than 1 × 10^6^), which could be detected or not detected as a function of the complexity of the matrix, are not considered. A fraction of the cloud water (200 mL) was filtered with a 0.22 µm nylon filter, and an aliquot was immediately frozen (referred to as INITIAL); the aliquot was incubated at 15 °C (CHEM 15) and 5 °C (CHEM 5) under the same conditions described in the following paragraph. Two fractions of cloud water with an endogenous microbial population were incubated in a sterilized Erlenmeyer flask at 15 °C (BIO + CHEM 15) and 5 °C (BIO + CHEM 5), with 200 rpm shaking, under dark conditions for 60 hours. Incubation time was chosen to maximize microbial transformations of dissolved organic matter. The temperatures were selected because they are representative of winter (5 °C) and summer (15 °C) conditions observed at the PUY summit under cloudy conditions^3^.

Each fraction was filtered with a 0.22 µm nylon filter and then prepared for analysis by ultrahigh-resolution FT-ICR mass spectrometry by using the method described by Zhao *et al*.^26^ and the previously detailed analysis of the same sample in Bianco *et al*.^27^ and Supplementary (S1). The high-resolution mass spectrometry analysis was performed using an FT-ICR mass spectrometer (Bruker) equipped with an electrospray ionization (ESI, Bruker) source set in negative ionization mode. The FT-ICR mass spectra were processed using the Composer software (Sierra Analytics, Modesto, CA): internal recalibration was performed, a peak list of signals, with S/N > 5, was generated, and the molecular formulas were assigned using the search criteria C_1–70_H_1–140_N_0–4_O_1–25_S_1_. The criteria described by Koch and Dittmar^59^ were applied to exclude formulas that do not occur abundantly in natural organic matter (NOM): DBE must have been an integer value, 0.2 ≤ H/C ≤ 2.4, O/C ≤ 1.0, N/C ≤ 0.5, S/C ≤ 0.2, 2 ≤ H ≤ (2 C + 2) and 1 < O ≤ (C + 2). All peaks were used without filtering by relative abundance because relative abundance was strongly dependent on the ionization capability and not only related to the concentration, especially in a complex matrix with sequential acquisition.

## Supplementary information

Supplementary Information
