# Publisher Correction: Effect of endogenous microbiota on the molecular composition of cloud water: a study by Fourier-transform ion cyclotron resonance mass spectrometry (FT-ICR MS)

**DOI:** 10.1038/s41598-019-53161-x

**Published:** 2019-11-06

**Authors:** Angelica Bianco, Laurent Deguillaume, Nadine Chaumerliac, Mickaël Vaïtilingom, Miao Wang, Anne-Marie Delort, Maxime C. Bridoux

**Affiliations:** 1 Université Clermont Auvergne, CNRS Laboratoire de Météorologie Physique, F-63000 Clermont-Ferrand, France; 2 CEA, DAM, DIF, F-91297 Arpajon, France; 3 Université Clermont Auvergne, CNRS, SIGMA-Clermont, Institut de Chimie de Clermont-Ferrand, F-63000 Clermont-Ferrand, France; 4 Laboratoire de Recherche en Géosciences et Energies (LaRGE), Departement of Physics, Université des Antilles, Pointe-à-Pitre, France

Correction to: *Scientific Reports* 10.1038/s41598-019-44149-8, published online 21 May 2019

The version of this Article previously published contained lower resolution images for Figures 1, 2, 3, 4, 5 and 6. This has now been corrected in the PDF and HTML versions of the paper.

