# Supplementary material for: Effect of endogenous microbiota on the molecular composition of cloud water: a study by Fourier-transform ion cyclotron resonance mass spectrometry (FT-ICR MS)
Source: Sci Rep. 2019 May 21;9:7663. doi: 10.1038/s41598-019-44149-8 (PMC6529453; doi:10.1038/s41598-019-44149-8)
Supplement: Supplementary file 1 — Supplementary Information [file 41598_2019_44149_MOESM1_ESM.docx]

**Effect of endogenous microbiota on the molecular composition of cloud water: a study by Fourier-transform ion cyclotron resonance mass spectrometry (FT-ICR MS)**

**Angelica Bianco^1,2^, Laurent Deguillaume^1*^, Nadine Chaumerliac^1^, Mickaël Vaïtilingom^1,4^, Miao Wang^1^, Anne-Marie Delort^3^, Maxime C. Bridoux^2*^**

*^1^ Université Clermont Auvergne, CNRS Laboratoire de Météorologie Physique, F-63000 Clermont-Ferrand, France.*

*^2^ CEA, DAM, DIF, F-91297 Arpajon, France*

*^3^ Université Clermont Auvergne, CNRS, SIGMA-Clermont, Institut de Chimie de Clermont-Ferrand, F63000 Clermont-Ferrand, France.*

*^4^ now at Laboratoire de Recherche en Géosciences et Energies (LaRGE), Departement of Physics, Université des Antilles*

Corresponding author: [maxime.bridoux@cea.fr](mailto:maxime.bridoux@cea.fr); [l.deguillaume@opgc.univ-bpclermont.fr](mailto:l.deguillaume@opgc.univ-bpclermont.fr)

**S(1) Sample preparation for FT-ICR MS analysis**

The Strata-X (Phenomenex) cartridges (1 g of sorbent contained in TEFLON^®^ tubes) were used for solid phase extraction and conditioned with consecutive application of 3 mL isopropanol, 6 mL acetonitrile, 6 mL methanol containing 0.1 % of formic acid and 6mL of ultrapure (MilliQ) water containing 0.1% of formic acid. TEFLON® tubes were used to avoid contamination released by plastic SPE tubes, creating a high background. Then 50 mL of cloud water with pH adjusted at 4.5 with concentrated formic acid was applied at the rate of 1 mL min^-1^ to the cartridge. The cartridges were then rinsed with 4 mL MilliQ acidified water to remove the inorganic salts remaining in the dead volume of the cartridge, these salts could reduce sensitivity in analysis by electrospray ionization (ESI) source. Cartridges were subsequently dried and analytes were eluted with 2.0 mL of acetonitrile/methanol/water (45/45/10) at pH 10.4. Solutions were stocked at 4°C in glass vial with TEFLON® cap until analysis.

Low molecular weights compounds (<100 Da) are expected to be lost in the rinsing and drying step of the extraction, as previously reported by Zhao et al. ^1^

Sample blank was prepared by spreading 100 mL of ultrapure water (MilliQ^®^) on sterile cloud aqueous phase collector. Water was collected in a clean glass bottle; pH was adjusted to 4.5 with formic acid and the blank sample was extracted by SPE with the same method used for real cloud water samples. Blank signal acquisition was performed with FT-ICR MS using the same condition described for the samples. Mass signals found in the blank were excluded (not subtracted) in Composer software.

All solvents were of HPLC grade or higher.

**S(2) Determination of carbon oxidation state (OSC):** the carbon oxidation state (OSC) is an ideal metric to measure the degree of oxidation of organic species in the atmosphere ^2^ and it is calculated using Equations S1 and S2 :

$OSC=\sum_{i} {OS}_{i}\times\frac{n_{i}}{n_{c}}$ (S1)

$OSC=2\times\frac{O}{C}-\frac{H}{C}-3\frac{N}{C}-2\frac{S}{C}$ (S2)

**S(3) Determination of aromaticity index (AI):** AI is determined as the ratio between DBE_AI(mod)_ and the number of carbon atoms C (Equations S3 and S4), as described by Melendez-Perez et al. ^3^ :

${DBE}_{AI(mod)}=1+\frac{1}{2}\times(2C-H-O-2S-N)$ (S3)

$AI=\frac{{DBE}_{AI(mod)}}{C}= \frac{1+\frac{1}{2}\times(2C-H-O-2S-N)}{C}$ (S4)

Note that if DBE_AI_ ≤ 0 or C_AI_ ≤ 0, then AI = 0. AI measures C-C double bond density and also integrates the contribution of π-bonds by heteroatoms.

**S(4) Determination of CHO index:** CHO index is calculated using equation S5:

$CHO index=\frac{2\times O-H}{C}$ (S5)


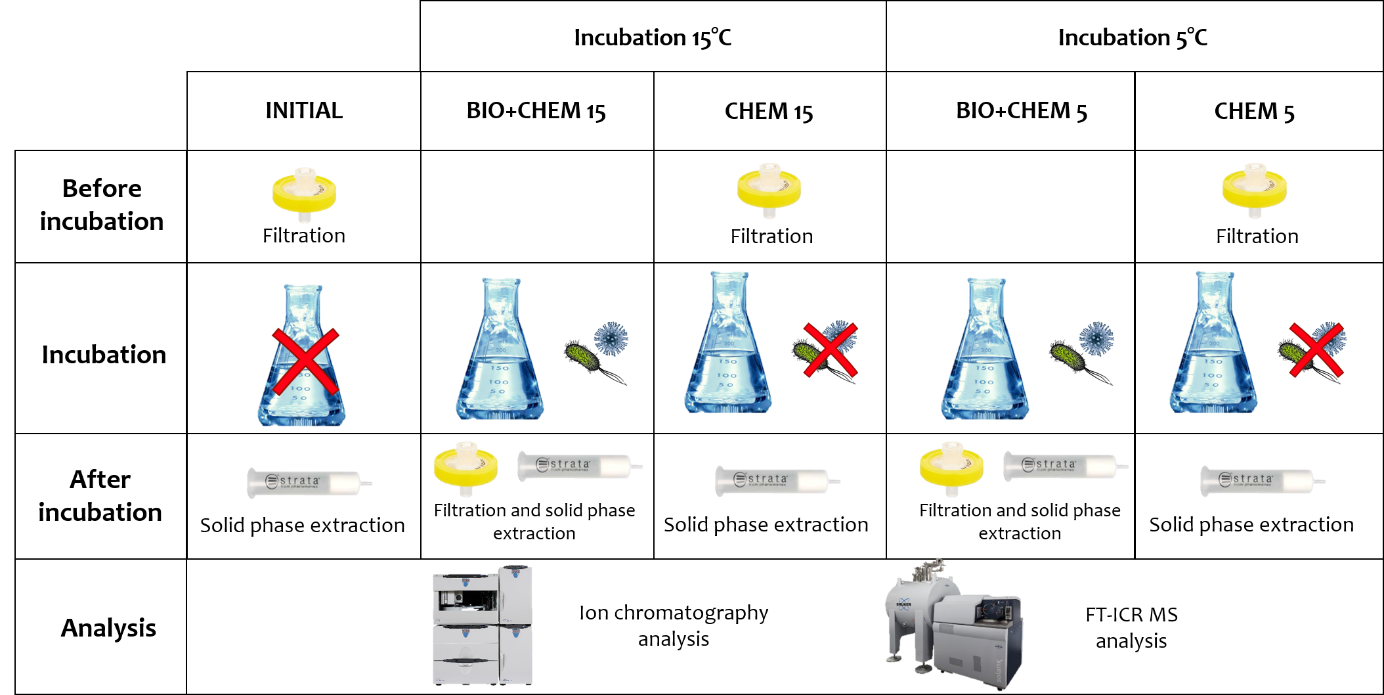


**Figure S1:** Various incubation tests performed considering filtration or not and changing the temperature.


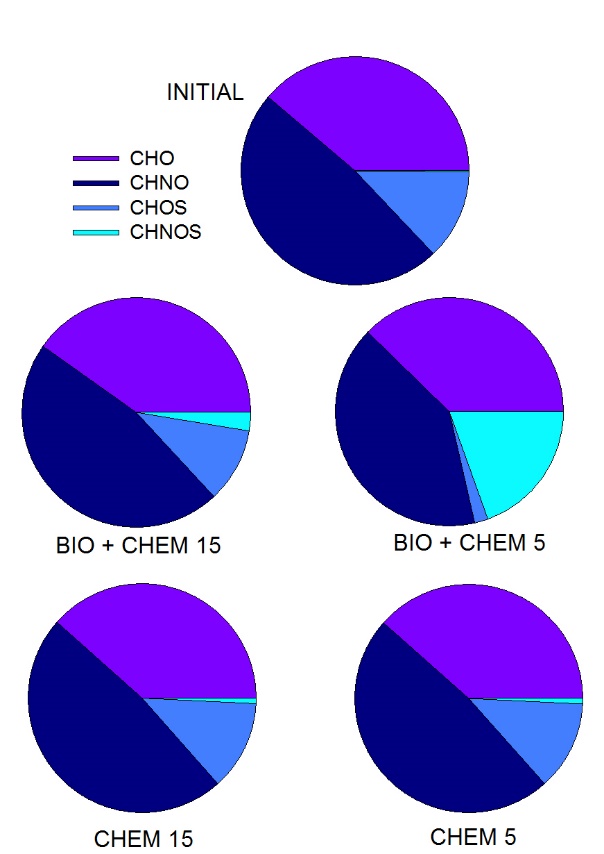


Figure S2: Pie plots of relative abundance in number of assigned molecular formula of CHO, CHNO, CHOS and CHNOS in INITIAL and in the incubated fractions at 15 and 5°C with (“BIO+CHEM”) and without microorganisms (“CHEM”).


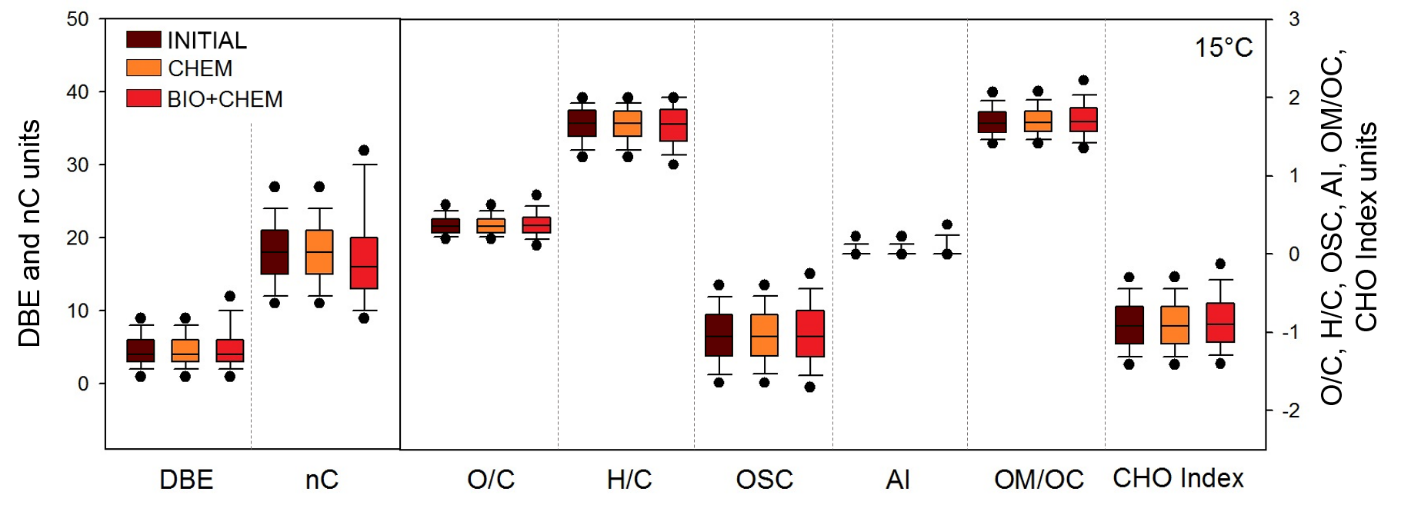


Figure S3: Average values of DBE, nC, O/C, H/C, OSC, AI, OM/OC and CHO Index calculated for all the assigned molecular formula for INITIAL, CHEM and BIO+CHEM for incubation at 15°C. The bottom and top lines of the box correspond to the 25^th^ and 75^th^ percentiles, respectively. The middle line represents the median. The ends of the whiskers are the 10^th^ and 90^th^ percentiles, and the filled circle is an outlier. The y-left scale shows the values of DBE and nC, the y-right scale the values of O/C, H/C, OSC, AI, OM/OC and CHO Index.


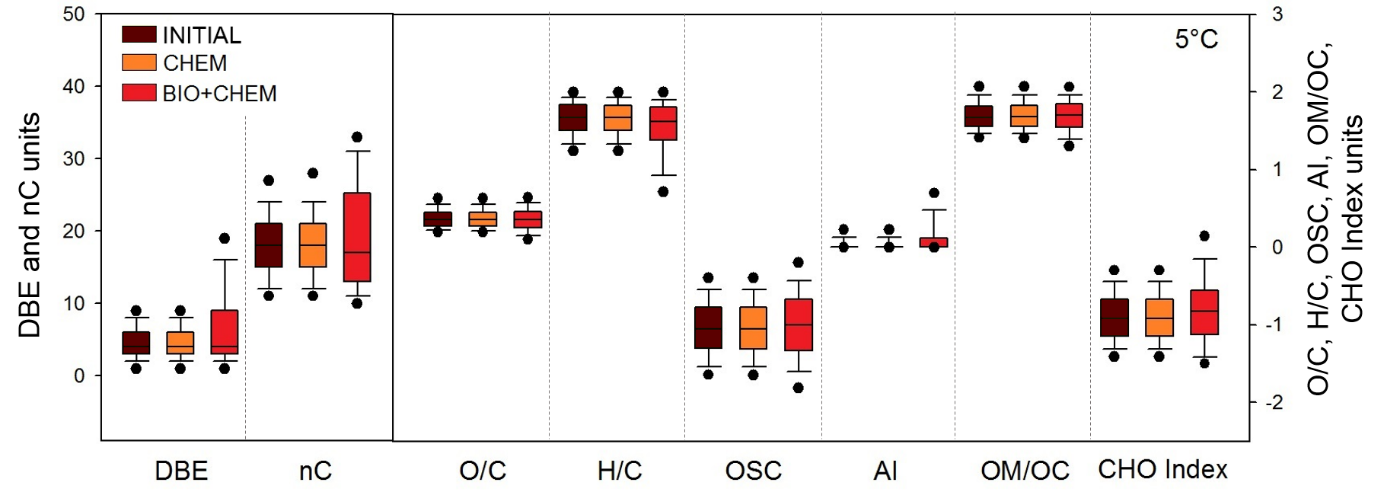


Figure S4: Average values of DBE, nC, O/C, H/C, OSC, AI, OM/OC and CHO Index calculated for all the assigned molecular formula for INITIAL, CHEM and BIO+CHEM for incubation at 5°C. The bottom and top lines of the box correspond to the 25^th^ and 75^th^ percentiles, respectively. The middle line represents the median. The ends of the whiskers are the 10^th^ and 90^th^ percentiles, and the filled circle is an outlier. The y-left scale shows the values of DBE and nC, the y-right scale the values of O/C, H/C, OSC, AI, OM/OC and CHO Index.


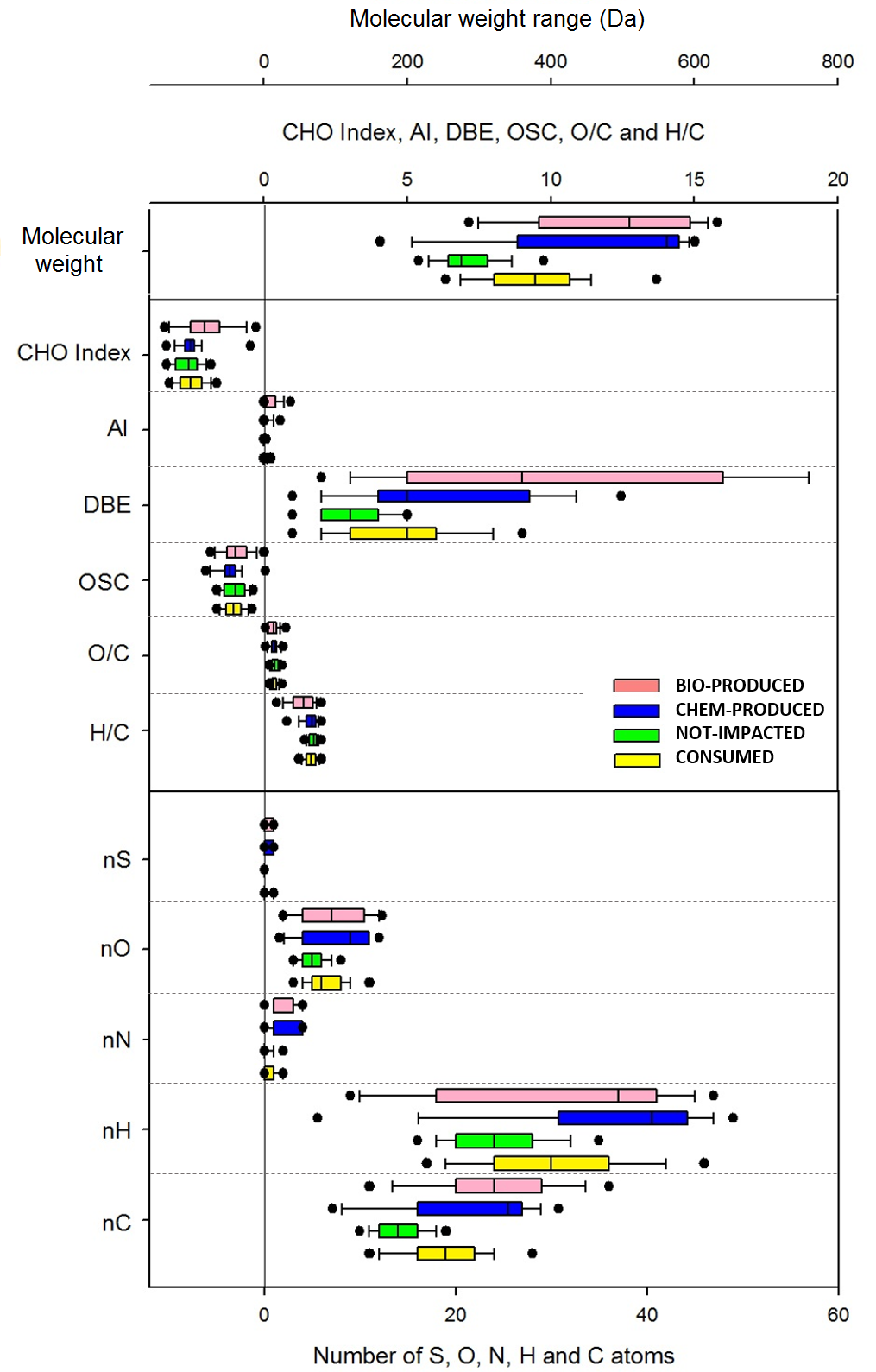


Figure S5: Box plots for CHO index, AI, DBE, OSC, O/C, H/C calculated for the different fractions (BIO-PRODUCED, CHEM-PRODUCED, NOT-IMPACTED, CONSUMED) reported in Figure 5, for incubation at 5°C. The bottom and top lines of the box correspond to the 25^th^ and 75^th^ percentiles, respectively. The middle line represents the median. The ends of the whiskers are the 10^th^ and 90^th^ percentiles, and the filled circle is an outlier. The y-right scale reports parameters; bottom x-scale reports values for nS, nO, nN, nH and nC; top x-scale the values for CHO index, AI, DBE, OSC, O/C, H/C, while upper x-scale reports values for mass weight.


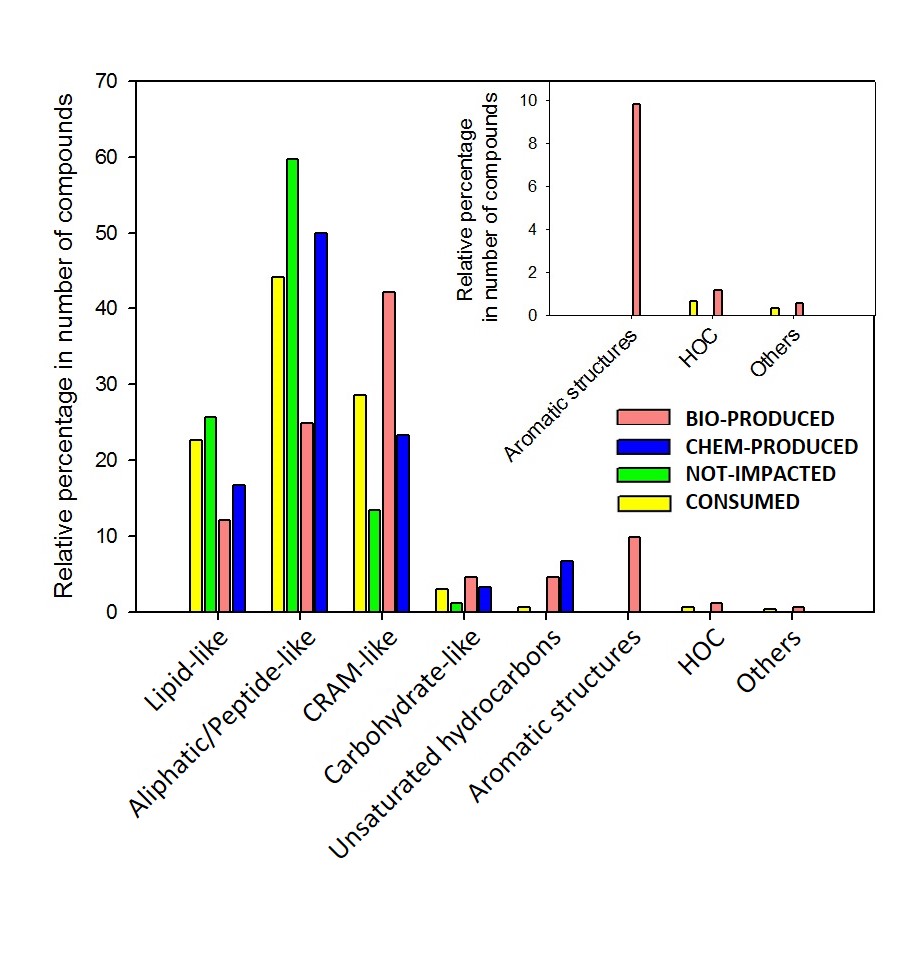


Figure S6: Number of total compounds and compounds contained in lipid-like, aliphatic/peptide-like and CRAM-like, carbohydrate-like, unsaturated hydrocarbons, aromatic structures and HOC in CONSUMED, NOT-IMPACTED, BIO-PRODUCED and CHEM-PRODUCED fractions for incubation at 5°C.


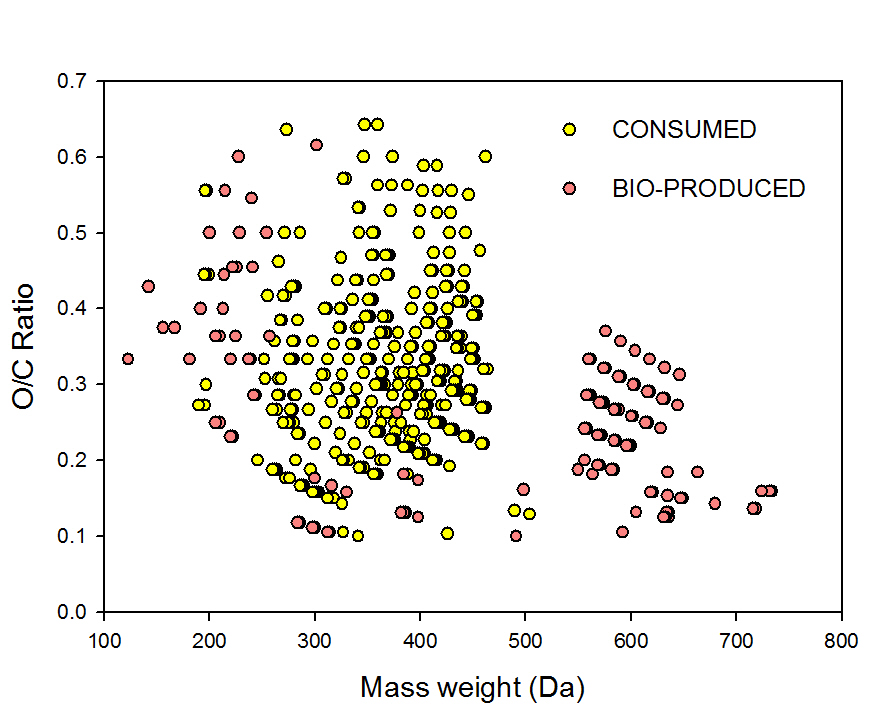


Figure S7: Comparison of O/C *vs* mass weight in CONSUMED and BIO-PRODUCED for incubation at 15°C for compounds contained in CRAM-like region.


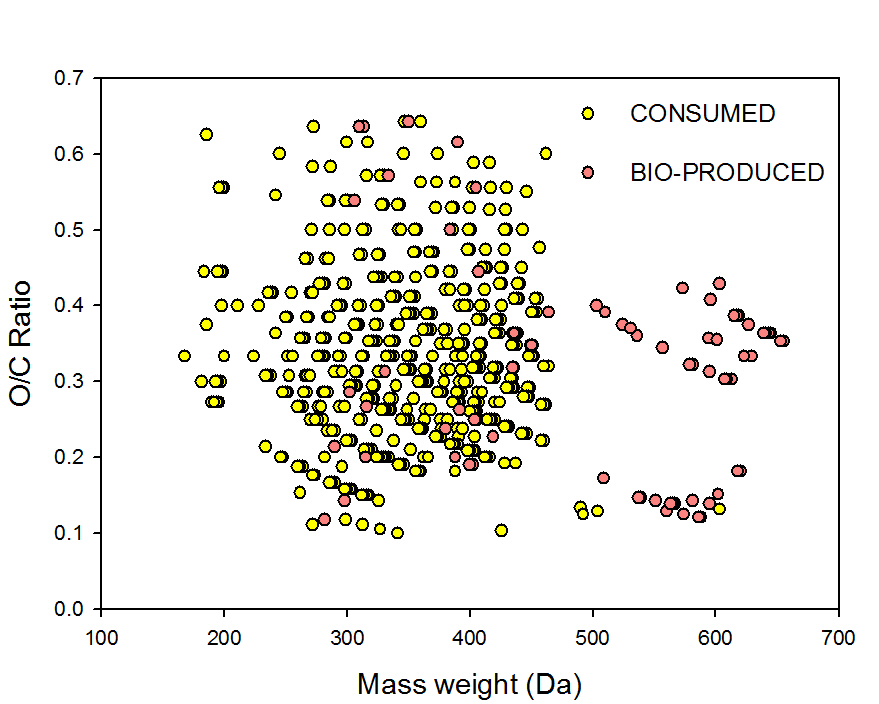


Figure S8: Comparison of O/C *vs* mass weight in CONSUMED and BIO-PRODUCED for incubation at 5°C for compounds contained in CRAM-like region.

| **Cloud parameters** | **Sampling time** | 2.50 PM - 7.20 PM | **Sampling day** | 01/06/2016 |
| --- | --- | --- | --- | --- |
|  | **Air-mass origin** | North | **Temperature during sampling** | 7.5°C |
|  | **Air-mass classification** | Marine | **LWC (Liquid Water Content)** | 0.2 g m^-3^ |
|  | **pH** | 4.6 |  |  |
|  |  |  | **% of production (+) or degradation (-)** | |
|  |  | **INITIAL** | **BIO+CHEM 15** | **BIO+CHEM 5** |
| **Microbial parameters** | **Cellular concentration** | 8.6 10^4^ cells mL^-1^ | +4% | +1% |
|  | **ATP** | 2.24 10^3^ pmol L^-1^ |  |  |
|  | **ADP** | 5.7 10^2^ pmol L^-1^ | -61% | -17% |
|  | **ATP per cell** | 2.6 10^-5^ pmol cell^-1^ |  |  |
|  | **ADP/ATP** | 0.26 | -59% | -13% |
| **Chemical concentrations** | **DOC** | 1.9 mgC L^-1^ |  |  |
|  | **H_2_O_2_** | 3.7 µM | -57% | -33% |
|  | **Formaldehyde** | 3.3 µM | -30% | -19% |
|  | **Acetate** | 2.1 µM | -36% | -52% |
|  | **Formate** | 10.5 µM | -34% | -62% |
|  | **Oxalate** | 0.9 µM |  |  |
|  | **Sulfates** | 3.9 µM |  |  |
|  | **Nitrates** | 20.8 µM |  |  |
|  | **Chlorides** | 6 µM |  |  |
|  | **Sodium** | 13.4 µM |  |  |
|  | **Ammonium** | 6.2 µM |  |  |
|  | **Magnesium** | 3.3 µM |  |  |
|  | **Potassium** | 11.5 µM |  |  |
|  | **Calcium** | 12.7 µM |  |  |

**Table S1**: Bio-physico-chemical characterization of the cloud sample. Production (+) or degradation (-) of targeted compounds are also indicated for the two incubations experiments at 15 and 5°C.

| **Class** | **H/C** | **O/C** |
| --- | --- | --- |
| Lipid-like | 1.5<H/C≤2.0 | 0≤O/C≤0.3 |
| Aliphatic/peptide-like | 1.5<H/C≤2.2 | 0.3<O/C≤0.67 |
| CRAM-like structures | 0.67<H/C≤1.5 | 0.1≤O/C<0.67 |
| Carbohydrate-like | 1.5<H/C≤2.5 | 0.67<O/C<1.0 |
| Unsaturated hydrocarbons | 0.67<H/C≤1.5 | O/C<0.1 |
| Aromatic structures | 0.2≤H/C≤0.67 | O/C<0.67 |
| Highly Oxygenated Compounds (HOC) | 0.6<H/C≤1.5 | 0.67≤O/C≤1.0 |

**Table S2**: Stoichiometric ranges of VK classes. Compounds with H/C and O/C ratios not included in these 6 classes are regrouped in “Others” class.

|  | **Incubation at 15°C** | | | | | | **Incubation at 5°C** | | | | | |
| --- | --- | --- | --- | --- | --- | --- | --- | --- | --- | --- | --- | --- |
|  | **CONSUMED** | | | **BIO-PRODUCED** | | | **CONSUMED** | | | **BIO-PRODUCED** | | |
|  | **Number of**  **compounds** | **%** | **Average molecular weight** | **Number of**  **compounds** | **%** | **Average molecular weight** | **Number of**  **compounds** | **%** | **Average molecular weight** | **Number of**  **compounds** | **%** | **Average molecular weight** |
| **Total** | 1094 |  | 401 ± 85 | 266 |  | 427 ± 175 | 1716 |  | 378 ± 85 | 173 |  | 476 ± 125 |
| **Lipid-like** | 303 | 27.7 | 398 ± 79 | 44 | 16.5 | 540 ± 129 | 388 | 22.6 | 387 ± 81 | 21 | 12.1 | 333 ± 60 |
| **Aliphatic/peptide-like** | 430 | 39.3 | 432 ± 88 | 38 | 14.3 | 331 ± 152 | 758 | 44.2 | 397 ± 89 | 43 | 24.9 | 555 ± 96 |
| **CRAM-like structures** | 333 | 30.4 | 364 ± 63 | 119 | 44.7 | 468 ± 180 | 491 | 28.6 | 347 ± 68 | 73 | 42.2 | 504 ± 117 |
| **Carbohydrate-like** | 12 | 1.1 | 319 ± 33 | 15 | 5.6 | 235 ± 41 | 52 | 3.0 | 295 ± 35 | 8 | 4.6 | 337 ± 134 |
| **Unsaturated hydrocarbons** | 8 | 0.7 | 540 ± 143 | 39 | 14.7 | 383 ± 133 | 10 | 0.6 | 520 ± 136 | 8 | 4.6 | 527 ± 47 |
| **Aromatic structures** | 0 | 0.0 | 0 | 2 | 0.8 | 387 ± 1 | 0 | 0.0 | 0 | 17 | 9.8 | 386 ± 36 |
| **HOC** | 6 | 0.5 | 316 ± 32 | 9 | 3.4 | 269 ± 62 | 11 | 0.6 | 297 ± 34 | 2 | 1.2 | 307 ± 13 |
| **Others** | 2 | 0.2 |  | 0 | 0.0 |  | 6 | 0.3 |  | 1 | 0.6 |  |

Table S3: Number of compounds, relative percentage and average mass weight for each class of compounds for CONSUMED and BIO-PRODUCED fractions for incubations at 15 and 5°C.

1. Zhao. Y.. Hallar. A. G. & Mazzoleni. L. R. Atmospheric organic matter in clouds: exact masses and molecular formula identification using ultrahigh-resolution FT-ICR mass spectrometry. *Atmospheric Chemistry and Physics* **13.** 12343–12362 (2013).

2. Kroll. J. H. *et al.* Carbon oxidation state as a metric for describing the chemistry of atmospheric organic aerosol. *Nature Chemistry* **3.** 133–139 (2011).

3. Melendez-Perez. J. J.. Martínez-Mejia. M. J. & Eberlin. M. N. A reformulated aromaticity index equation under consideration for non-aromatic and non-condensed aromatic cyclic carbonyl compounds. *Organic Geochemistry* **95.** 29–33 (2016).
